# Acute tenosynovitis following an accidental injection of Bacille Calmette-Guérin (BCG) in a health care worker: A case report

**DOI:** 10.1016/j.infpip.2023.100332

**Published:** 2023-12-12

**Authors:** Mieko Tokano, Norihito Tarumoto, Kazuo Imai, Takuya Sekine, Yasuto Omura, Kosuke Uehara, Shigefumi Maesaki

**Affiliations:** a Department of Infectious Disease and Infection Control, Saitama Medical University, 38 Morohongo, Moroyama, Saitama, 350-0495, Japan; b Department of Allergy and Immunology, Faculty of Medicine, Saitama Medical University, 38 Morohongo, Moroyama, Saitama, 350-0495, Japan; c Department of Orthopaedic Surgery, Saitama Medical University, 38 Morohongo, Moroyama, Saitama, 350-0495, Japan

**Keywords:** Tenosynovitis, Mycobacterial infection, *Mycobacterium bovis* bacillus Calmette-Guérin (BCG), *Mycobacterium tuberculosis*, Accidental injection

## Abstract

**Case:**

A 36-year-old female healthcare worker with no past medical history, accidentally injected her flexed right middle finger with live attenuated *Mycobacterium bovis* bacillus Calmette-Guérin (BCG). Swelling and erythema around the injured area appeared two days after the needlestick injury. She was referred to the hospital and presented approximately nine days after self-inoculation. Surgical debridement was immediately performed. After 38 days, colonies were observed on cultures of the removed tissue on Ogawa's medium. This isolate was identified as *M. bovis* BCG by polymerase chain reaction (PCR) based on RD1 gene deletion. She had a history of BCG vaccination and her skin lesion appeared immediately after the accidental injection of *M. bovis* BCG. Therefore, in the differential diagnosis, the possibility that the lesion was an allergic reaction to BCG was considered. The subsequent culture results came back positive for *M. bovis* BCG and acute tenosynovitis caused by *M. bovis* BCG was diagnosed. The skin lesion was treated with anti-mycobacterial drugs and resolved.

**Discussion:**

The allergic reactions to BCG should be considered in the differential diagnosis of skin lesions following BCG vaccination. It is important to promptly submit a specimen for culture as delayed initiation of appropriate treatment can lead to a poor prognosis. In patients with accidental injection of *M. bovis* BCG, it is important to consider timely surgical excision and appropriate antimycobacterial therapy.

## Introduction

The *Mycobacterium bovis* bacillus Calmette-Guérin (BCG) strain is an attenuated derivative of a virulent strain of *Mycobacterium bovis* [1]. BCG vaccination has been used globally as prophylaxis against childhood tuberculous meningitis and miliary tuberculosis disease [2]. More recently, it has been used therapeutically as the most common form of immunotherapy for bladder cancer [3]. While the details of the mechanism of action remain unknown, *M. bovis* BCG is known to induce a robust innate immune response [3,4]. The intradermal or intramuscular injection of *M. bovis* BCG can lead to serious complications such as intractable ulcer formation [5]. However, the risks of the accidental injection of *M. bovis* BCG by healthcare workers are less clear due to the under reporting of events [6]. In addition, *M. bovis* BCG is difficult to differentiate from other strains of *M. bovis* and other members of the *M. tuberculosis* complex by conventional methods [1]. We report a case of acute tenosynovitis caused by *M. bovis* BCG in a healthcare worker after accidental injection with *M. bovis* BCG.

## Presentation of case

**Figure 1:**
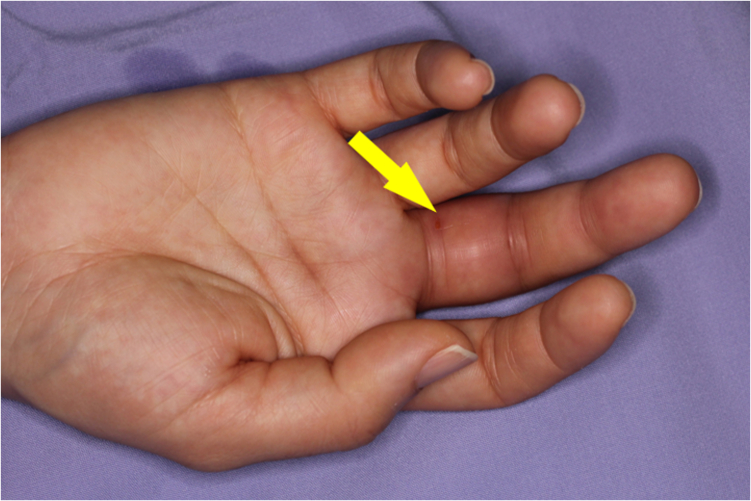
A preoperative volar view of the right index finger obtained approximately three days after the patient's arrival.

**Figure 2:**
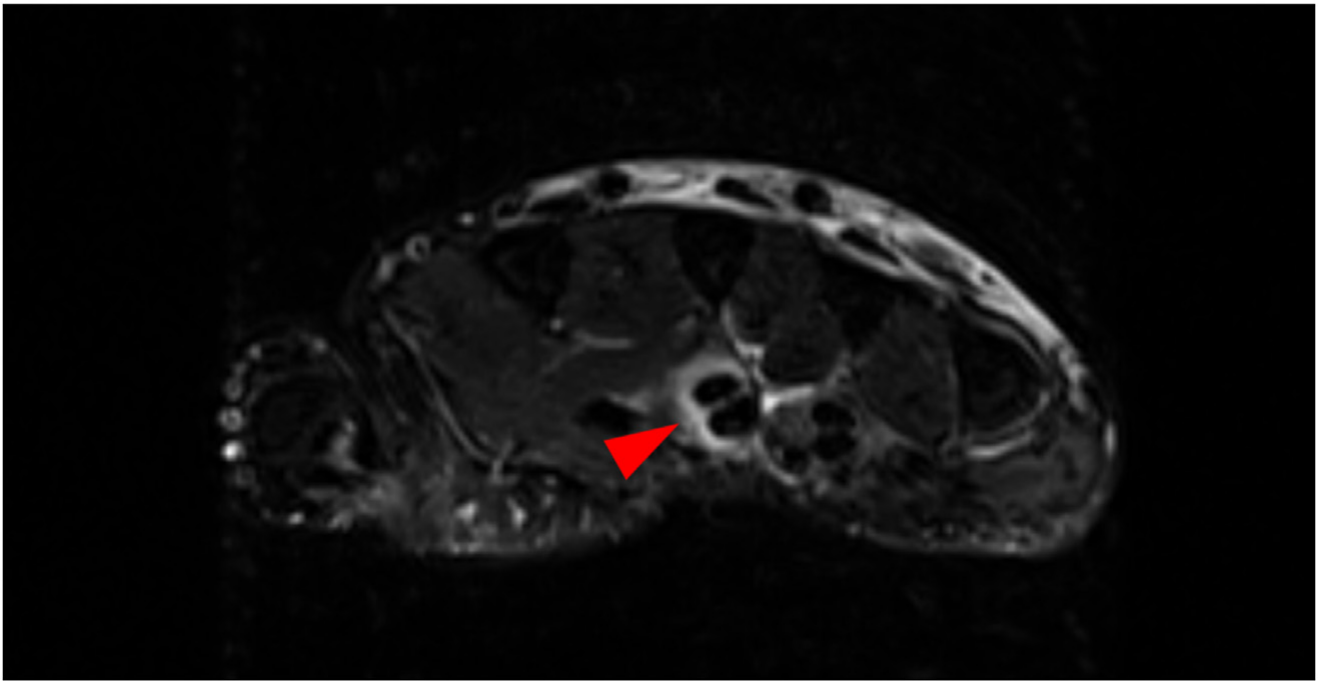
MRI on admission showed high-signal areas in the right middle flexor tendon and subcutaneous tissue of the right middle finger.

**Figure 3:**
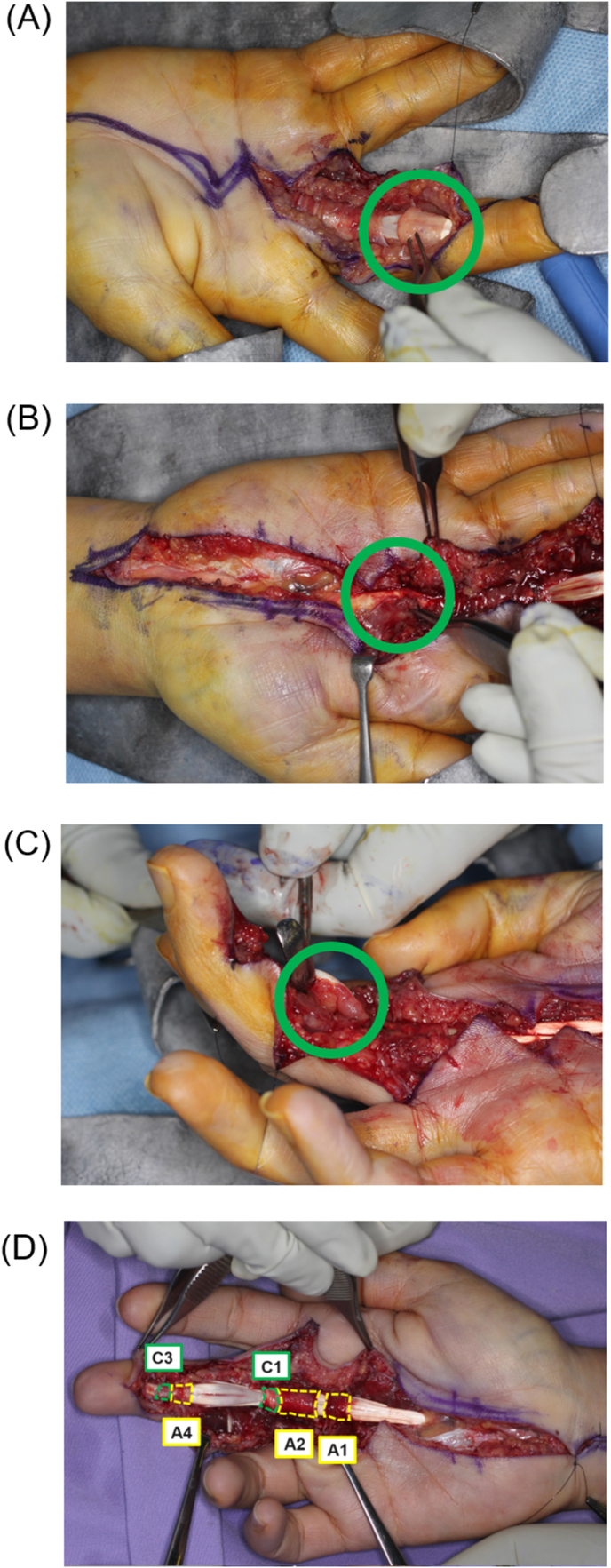
The A3 (A), A5, and palmar (B) aponeurosis pulleys were weakened and contaminated. (C) A synovial membrane similar to a grain of rice was attached around the deep finger flexor tendon. (D) The appearance of the finger following tenosynovectomy.

A 36-year-old female healthcare worker with no past medical history accidentally injected her flexed right middle finger with live attenuated *M. bovis* BCG (Immunobladder intravesical, Japan BCG Laboratory, Tokyo). She had a history of BCG vaccination. She adjusted the *M. bovis* BCG solution for bladder instillation according to the manufacturer's instructions, and while attempting to recap the needle, it slipped and pricked the ventral side of her finger. She did not experience any bleeding. Swelling and erythema around the injured area appeared two days after the needlestick injury. She noted that these skin symptoms progressed and she was treated with 1% rifampicin ointment. Rifampicin ointment may be used for skin lesions at the site of BCG vaccination [7]. However, her symptoms worsened, and the movable range of the joint portions became limited. She was referred to our hospital and arrived approximately nine days after self-inoculation. At presentation, physical examinations demonstrated that she was in good general health. Her finger demonstrated Kanavel signs (slight flexed posture, fusiform swelling over the affected tendon, tenderness over the affected tendon, pain on passive extension of the affected finger), which were consistent with acute suppurative flexor tenosynovitis (Figure 1 A, B). There was no visible necrosis of the soft tissues or discharge of pus observed on the surface of the body. She had no history of fever or swelling in any other part of the body. No abnormal data were seen in complete blood cell counts and serum chemistry. Radiographs showed no abnormalities. Magnetic resonance imaging (MRI) showed high-signal areas in the right middle flexor tendon and subcutaneous tissue of the right middle finger on short inversion time inversion recovery (STIR) sequence, suggesting tenosynovitis and cellulitis, respectively (Figure 2). Surgical debridement was immediately performed. The A3, A5, and palmar aponeurosis pulleys were excised because they were weakening and contaminated (Figure 3 A-D). A histological examination showed no findings characteristic of tuberculosis. Special staining (Ziehl-Nielsen staining) for acid-fast mycobacteria of the contaminated fluid collected from the wound during surgery was also negative. A direct smear microscopy examination with Ziehl-Nielsen staining was negative. The culture of the contaminated fluid collected during surgery using the Mycobacteria Growth Indicator Tube (BD BACTEC™ MGIT™ 960) (Becton Dickinson, Franklin Lakes, USA) was positive after 16 days. Furthermore, after 38 days, colonies were observed in tissue cultures on Ogawa's medium (Kyokuto Pharmaceutical, Tokyo, Japan). The colonies were susceptible to streptomycin, isoniazid, rifampicin, ethambutol, kanamycin, enviomycin, ethionamide, cycloserine, para-aminosalicylic acid, and levofloxacin, according to the Clinical and Laboratory Standards Institute (CLSI) Guidelines (document M45). The acid-fast bacillus detected in culture was identified as the *M. tuberculosis* complex using MALDI-TOF MS (Bruker, Germany, Filamentous Fungi Library 1.0). We performed polymerase chain reaction (PCR) to confirm that the strain of the *M. tuberculosis* complex was *M. bovis* BCG. To prepare the template DNA for PCR, bacterial cells from a single colony were suspended in sterile distilled water and lysed at 98°C for 5 min. Genomic DNA was extracted using UltraClean Microbial (QIAGEN, Venlo, Netherlands) according to the manufacturer's protocol. PCR amplicons were generated by TaKaRa SPPD STA (Takara Bio Inc., Gunma, Japan), and thermal cycling was carried out under the following conditions: 98°C for 2 min, followed by 40 cycles at 98°C for 5 s, 55°C for 15 s, 72°C for 10 s, with a final extension at 72°C for 3 min. PCR products were analyzed using 1% (w/v) agarose gel stained with ethidium bromide. The ET1 (AAGCGGTTGCCGCCGACCGACC), ET2 (CTGGCTATATTCCTGGGCCCGG), and ET3 (GAGGCGATCTGGCGGTTTGGGG) primers were used, as previously described [1]. ET1 and ET3 yielded a 190-bp product in the patient's isolate and the *M. bovis* BCG solution (Immunobladder intravesical, Japan BCG Laboratory, Tokyo). ET2 and ET3 yielded no product. In contrast, ET2 and ET3 yielded a 150-bp product in isolates of patients under treatment for pulmonary tuberculosis. Based on these results, her isolate was identified as *M. bovis* BCG. The patient was treated with anti-tuberculosis drugs (rifampin, isoniazid, and ethambutol daily). After 2 months, ethambutol was stopped after the resolution of the skin lesion. Anti-mycobacterial treatment (rifampin, isoniazid) was continued for the next 7 months.

## Discussion

Despite the use of *M. bovis* BCG for intravesical injection or vaccination and the frequency of accidental needlestick injury in healthcare workers, accidental injection of *M. bovis* BCG in healthcare professionals is rare [8,9]. Ten cases of hand infections related to *M. bovis* BCG preparation in healthcare workers have been reported (Table I) [6,[9], [10], [11], [12], [13], [14], [15], [16], [17]]. Five of the 10 cases involved intravesical injection, 4 involved vaccination, and 1 involved a laboratory-acquired infection. Culture tests were positive in only 3 of the 10 cases. The time from injury to the onset of symptoms ranged from a few hours to several months. Although the BCG vaccination history was unknown for many of the past cases, the BCG vaccination history may be related to the time from injury to the onset of symptoms. In our case, the patient had a history of BCG vaccination, and the time from injury to the onset of symptoms was very short. Because of the rapid onset of her symptoms, in the differential diagnosis, we initially had to consider the possibility that her lesion developed as an allergic reaction to *M. bovis* BCG. Based on the positive result of the tissue culture, we confirmed that her tenosynovitis was caused by *M. bovis* BCG. It is believed that the reason for the histological examination showing no findings characteristic of tuberculosis was that there had not been enough time elapsed since she was accidentally injected. Genetic testing alone cannot distinguish between dead and viable bacteria. It is known to be viable when a colony can be confirmed in the culture. Therefore, until the definitive diagnosis was made after three weeks of waiting for the culture results, she had no choice but to undergo observation while taking cephalexin orally. In previously reported cases, all cases with positive antimicrobial test results or pathology findings required surgery. In previous cases, the delayed initiation of treatment resulted in the need for reoperation. Fortunately, she underwent surgery approximately three days after she arrived and received anti-mycobacterial treatment three weeks after she arrived, and her skin lesion resolved.

**Table I:** Reported cases of accidental injection of BCG

Year, country	Age,Sex	Cause of injury	Time from injury to symptom onset	Basis for the diagnosis	Treatment	Reference
1956, Finland	32, F	vaccination	1 month	ND	none	[10]
1982, France	45, F	microbiological research (laboratory-acquired infections)	several weeks	culture, pathology	debridement, isoniazid, rifampin, and ethambutol for 12 months	[11]
1984, England	32, F	vaccination	2 months	culture	debridement, isoniazid and rifampin	[12]
1996, USA	29, M	intravesical injection	24 hours	direct smear	debridement, isoniazid and rifampin for 8 months	[13]
1998, Germany	59, F	vaccination	several days	direct smear, pathology	debridement, rifampin, ethambutol, and streptomycin for 6 months	[14]
1999, Japan	38, F	intravesical injection	2 weeks	pathology	debridement, isoniazid and rifampin for 6 months	[15]
2008, USA	42, F	intravesical injection	2–3 weeks	no pathogens were detected.	debridement, rifampin, ethambutol, levofloxacin, and amikacin for 6 months	[16]
2008, Croatia	42, F	intravesical injection	2 weeks	culture	debridement rifampin, isoniazid, ethambutol, and streptomycin for 9 months	[17]
2011, India	ND, F	vaccination	5 weeks	ND	none	[6]
2013, USA	29, F	intravesical injection	3 hours	no pathogens were detected.	debridement, isoniazid, rifampin, ethambutol, and pyrazinamide for 6 months	[9]

Abbreviations: ND no data.

Previously reported cases, including ours, were treated with long-term anti-mycobacterial treatment. In previously reported cases, medications were administered for 6–12 months. Treatment differs between *M. tuberculosis* and *M. bovis* BCG because *M. bovis* BCG, unlike *M. tuberculosis*, is inherently resistant to pyrazinamide. Therefore, it should be treated with an antimycobacterial drug other than pyrazinamide. A definitive diagnosis of *M. bovis* BCG is important, but it is difficult to discriminate between the *M. tuberculosis* complex and *M. bovis* BCG using conventional laboratory methods and genomic analysis is required. One region of difference, designated RD1, was shown to be absent in *M. bovis* BCG strains and present in *M. bovis* strains and *M. tuberculosis* strains. For a definitive diagnosis of *M. bovis* BCG infection, the deletion of RD1 must be confirmed by PCR. In strains without RD1, ET1, and ET3 bind and amplify a 190-bp region. In strains with RD1, these primers bind, but the 9,650-bp sequence is too large to efficiently amplify [1]. In our case, ET1 and ET3 yielded a 190-bp product in the patient's isolate. When we encounter patients with suspected infection caused by *M. bovis* BCG, even if it is identified as *M. tuberculosis* complex using common laboratory reagents, we need to identify the species by PCR.

Accidental injection of *M. bovis* BCG, as in our case, can result in a severe occupational injury. Therefore, healthcare workers must be extremely careful when handling *M. bovis* BCG solutions. While it is advised to avoid needle-recapping, there may be situations where it cannot be avoided. For example, healthcare workers could use a needle-recapping device when handling syringes containing *M. bovis* BCG solution to prevent accidental injection. Furthermore, used needles should be promptly discarded in a sharps bin.

## Conclusion

Accidental injection of *M. bovis* BCG may cause rapid suppurative mycobacterial tenosynovitis. If a patient has a history of BCG vaccination, allergic reactions to *M. bovis* BCG should also be included in the differential diagnosis. Delayed initiation of treatment can lead to poor prognosis, so in patients with accidental injection of *M. bovis* BCG, it is important to submit the specimen promptly for culture and consider timely surgical excision and appropriate antimycobacterial therapy.

## Informed consent statement

Written informed consent was obtained from the patient for the publication of this case report and accompanying images.

## Conflicts of interest

The authors have no conflicts of interest in association with the present study.
